# Global Insights on Prehospital Stroke Care: A Comprehensive Review of Challenges and Solutions in Low- and Middle-Income Countries

**DOI:** 10.3390/jcm13164780

**Published:** 2024-08-14

**Authors:** Elvan Wiyarta, Marc Fisher, Mohammad Kurniawan, Rakhmad Hidayat, Iskandar Purba Geraldi, Qaisar Ali Khan, I Putu Eka Widyadharma, Aliena Badshah, Jeyaraj Durai Pandian

**Affiliations:** 1 Department of Neurology, Faculty of Medicine, Universitas Indonesia-Dr. Cipto Mangunkusumo National Hospital, Central Jakarta, Jakarta 10430, Indonesia; m.kurniawan@ui.ac.id (M.K.); rhidayat.md@gmail.com (R.H.); 2 Beth Israel Deaconess Medical Center, Boston, MA 02215, USA; mfisher5@bidmc.harvard.edu; 3 Cengkareng General Hospital, West Jakarta, Jakarta 11730, Indonesia; 4 Department of Medicine, Khyber Teaching Hospital, Peshawar 25120, Pakistanaliena.badshah@kmc.edu.pk (A.B.); 5 Department of Neurology, Faculty of Medicine, Universitas Udayana, Bali 80361, Indonesia; 6 Christian Medical College, Ludhiana 141008, Punjab, India; jeyaraj.pandian@cmcludhiana.in

**Keywords:** prehospital stroke care, low- and middle-income countries, stroke awareness, stroke management, healthcare infrastructure, mobile stroke units, telemedicine, thrombolytic therapy, public health education, emergency medical services

## Abstract

Stroke is a leading cause of disability and mortality worldwide, and it disproportionately affects low- and middle-income countries (LMICs), which account for 88% of stroke fatalities. Prehospital stroke care delays are a crucial obstacle to successful treatment in these settings, especially given the limited therapeutic window for thrombolytic treatments, which may greatly improve recovery chances when initiated early after stroke onset. These delays are caused by a lack of public understanding of stroke symptoms, sociodemographic and cultural variables, and insufficient healthcare infrastructure. This review discusses these issues in detail, emphasizing the disparities in stroke awareness and reaction times between locations and socioeconomic classes. Innovative options for reducing these delays include the deployment of mobile stroke units and community-based educational campaigns. This review also discusses how technology improvements and personalized educational initiatives might improve stroke awareness and response in LMICs. The primary goal is to give a thorough assessment of the challenges and potential remedies that might serve as the foundation for policy reforms and healthcare improvements in LMICs, eventually improving stroke care and lowering disease-related mortality and disability.

## 1. Introduction

Stroke is a non-communicable neurological disease with a high prevalence [1]. Throughout the world, stroke is one of the main causes of disability. Apart from this, stroke is also known to be the second largest cause of death worldwide [2,3]. Although in some countries there has been a reduction in stroke incidence of up to 42%, population-based research shows that there has been a 100% increase in small countries [4]. In addition, around 87% of disability-related stroke events between 1970 and 2010 occurred mainly in low- and middle-income countries (LMICs) [5,6]. This is seven times higher than in countries with higher incomes [5,6]. Stroke mortality rates in high-income countries are known to have decreased [3,7]. However, a different picture is seen in developing countries, with the burden caused by stroke increasing in recent years and predicted to continue to increase [3,7].

A stroke is a medical emergency characterized by a disruption or decrease in blood flow in the brain, which results in decreased perfusion in brain tissue [3]. There are two major types of strokes: ischemic and hemorrhagic [3]. Ischemic strokes occur when a blood clot blocks or narrows an artery leading to the brain, reducing blood flow and oxygen supply to brain tissue [3]. Hemorrhagic strokes, on the other hand, occur when a blood vessel in the brain bursts, leading to bleeding in or around the brain. These two types of strokes have different pathophysiological mechanisms and treatment approaches. Ischemic strokes are typically treated with thrombolytic therapy to dissolve the clot, whereas hemorrhagic strokes often require surgical intervention to stop the bleeding and relieve pressure on the brain [8].

Treatment with thrombolytics helps to restore blood flow and reduces damage to the brain, thereby increasing the chances of an improved recovery [8]. However, one of the biggest challenges is that this therapy can only be given for a fixed period. The “time is brain” concept, which was introduced two decades ago, also emphasizes that time is crucial in treating acute stroke, especially with the use of thrombolysis and endovascular therapy [9]. If there is a delay, the patient will lose the opportunity for therapy that might be beneficial [10]. Observational studies using the Modified Rankin Scale (mRS) as the outcome measure show that 1 min faster treatment using thrombolysis is associated with an increase of 1.8 days of healthy life and a decrease of 15 min to 1 month of disability-free life [11].

Many factors can certainly influence a patient’s delay in seeking help when experiencing an acute stroke. Conditions such as poor knowledge regarding stroke, its potential treatment, and socioeconomic conditions influence prehospital delays. Research also finds that there are disparities between high-income countries and LMICs, with research showing that in Europe and North America, the population largely has a good knowledge of stroke [12,13,14,15,16]. Research conducted by Aguirre et al. also found that in a pooled meta-analysis from 19 LMICs, there were only 48.4% of patients who arrived at the hospital within 24 h after the onset of stroke [1]. This is in contrast to the latest pooled analysis in certain countries, which had an onset-to-treatment time of only 233 min [17]. Prehospital delays are one of the reasons causing mortality and long-term physical disability in patients [17]. Research also finds that the delay time is most influenced by the time from the stroke to the first encounter with medical personnel [18]. Therefore, action is needed to reduce delays that occur in initial assessment and treatment, especially prehospital delays [1].

This study aims to provide a comprehensive review of the challenges and solutions in prehospital stroke care in LMICs. By analyzing the existing literature, this review seeks to identify key factors contributing to delays in prehospital stroke care and propose actionable strategies to mitigate these delays. The goal is to enhance understanding and facilitate the development of effective interventions to improve stroke outcomes in LMICs, ultimately reducing the global burden of stroke-related disability and mortality.

## 2. Challenges in Prehospital Delays for Stroke Care in LMICs

**Figure 1:**
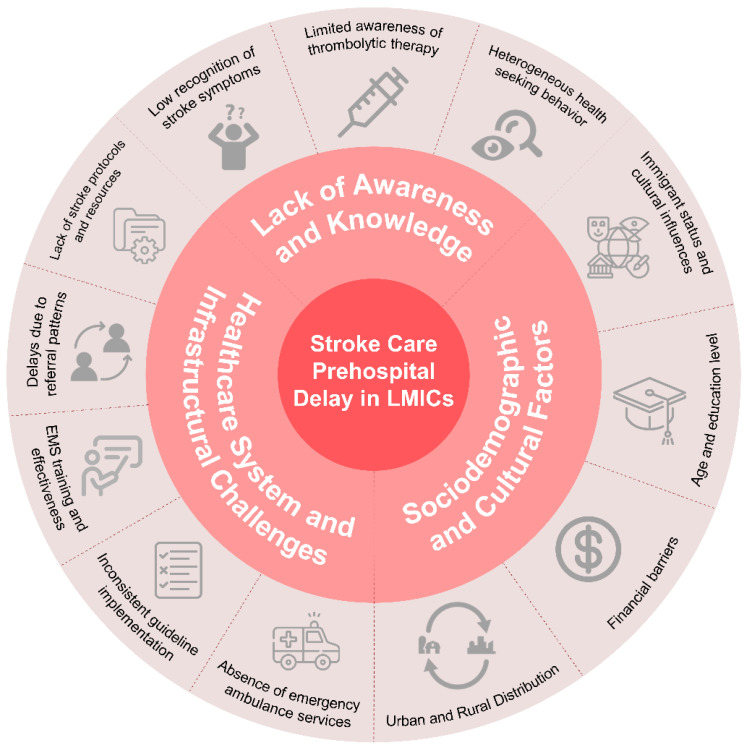
Factors contributing to prehospital delays in stroke care in low- and middle-income countries. The central section highlights three primary categories: lack of awareness and knowledge, sociodemographic and cultural factors, and healthcare system and infrastructural challenges. Each category contains specific contributing factors. Lack of awareness and knowledge includes low recognition of stroke symptoms, limited awareness of thrombolytic therapy, and heterogeneous health-seeking behavior. Sociodemographic and cultural factors cover immigrant status and cultural influences, age and education level, financial barriers, and urban–rural distribution. Healthcare system and infrastructural challenges encompass the absence of emergency ambulance services, inconsistent guideline implementation, delays due to referral patterns, lack of protocols, and undertrained emergency medical services.

Understanding the factors that lead to prehospital stroke care delays in LMICs is critical for creating effective solutions. These delays are caused by a variety of circumstances, including a lack of awareness and information on stroke symptoms among general people and medical professionals [3,17,19]. Furthermore, sociodemographic and cultural factors play an important role, as differences in education, economic status, and cultural attitudes might limit timely medical intervention [3,12,17]. The healthcare system, with its infrastructure and resource limits, exacerbates the delays [3,17]. This section aims to discuss the multifaceted barriers to timely stroke treatment in LMICs (Figure 1), providing a foundation for targeted strategies to improve stroke outcomes.

### 2.1. Lack of Awareness and Knowledge

Various studies have found that there is a large gap in knowledge of stroke symptoms and appropriate emergency responses, as summarized in Table 1. In India, research found that the prehospital delay time in patients with suspected stroke was 716 min (11 h, 56 min) [18]. In this study, around 84% of patients or their primary caregivers had never heard of stroke or knew the symptoms of stroke [18]. The substantial prehospital delay time of almost 12 h is known to occur due to poor recognition of the early symptoms of stroke and low perception of the dangers of arriving late at the hospital for stroke patients [18]. This study also suggests that a lack of awareness of stroke symptoms may contribute to delayed treatment [18]. Another study in India conducted by Ashraf et al. also obtained similar results, with only 25.3% of patients arriving within 4 h of stroke onset [20]. Although the recognition of stroke symptoms in this study was better than in previous studies, there was still a significant delay in the patient’s arrival at the stroke unit [20].

Research conducted in Pakistan found that 72.5% of patients arrived in less than 4.5 h. This study found that delays occurred in patients with low stroke severity [17]. Meanwhile, a study conducted in Ghana found that patients experiencing delays were caused by low levels of awareness of the early symptoms of stroke [21]. These patients generally sought other treatment, such as self-medication, before arriving at the hospital [21]. Delays in contacting emergency medical services (EMS) or coming to the emergency room (ER) were related to poor knowledge of stroke, such as if the stroke symptoms experienced were only temporary or unimportant and suspecting that the symptoms were caused by something else [12]. Research conducted by Iqbal et al. in 2016 found that around 92.6% of patients arrived more than 4.5 h after stroke onset [22]. This is associated with the level of public awareness and in referring doctors regarding the window time for administering thrombolytic therapy [22].

Apart from this, cultural beliefs and practices like assuming that strokes are caused by spiritual things and retribution from God that cannot be handled medically also influence patterns in seeking healthcare. The effect of this belief is that patients generally seek herbal or traditional treatment or even try to combine these while in the hospital. It is presumed that sociocultural beliefs and practices result in limited outpatient attendance for follow-up after patients are discharged, with such patients seeking local herbal remedies or prayer camps [21]. Religious or cultural beliefs are also a barrier, even when patients are having a stroke. Half of stroke patients in Bolivia do not go to a hospital or doctor [23]. Research by Hertz et al. explains that knowledge or awareness of stroke symptoms and their relationship with health-seeking behavior is heterogeneous [16]. A high level of knowledge and perception of stroke risk was not associated with hospital selection preferences when a stroke symptom occurred [16].

In Pakistan, a study by Ali et al. found that 72.5% of patients arrived at the hospital within 4.5 h [17]. However, 16.3% of patients were indirectly taken to the hospital’s emergency clinic and neurology clinic [17]. These 16 patients were initially admitted to another hospital after stroke onset, with only four knowing about thrombolytic therapy. With 92.6% arriving late at the hospital using private vehicles [17]. Although health-seeking behavior is heterogeneous, research conducted by Shephard et al. found that higher stroke knowledge was associated with shorter prehospital delays [24]. In conditions where symptoms are considered not serious, there is an increase in delay in the decision-making process [24].

**Table 1:** Awareness and knowledge barrier in low- and middle-income countries.

Challenges of Prehospital Delay in Stroke Care	Country
Low recognition and perception of stroke symptoms	India [18,20]
Low awareness of thrombolytic therapy and acute stroke referrals among the public and doctors	Pakistan [17,22]
The belief that strokes are caused by spiritual reasons, leading to self-treatment through herbal practices or prayer camps	Ghana [21]
High knowledge and perception of stroke not influencing hospital selection preferences	Tanzania [16]

### 2.2. Sociodemographic and Cultural Factors

Apart from awareness and knowledge factors, sociodemographic and cultural factors also play a role in prehospital delay in LIMCs, as shown in Table 2. Pallangyo et al. from Tanzania found that those aged over 55 years had superior knowledge of the warning signs of stroke [25]. Regarding education level, respondents with secondary education or higher, who had hypertension, and who had a family history of stroke showed higher knowledge scores regarding the signs and risk factors of stroke compared to those without [25]. Kakame et al. in Uganda found that 91% of patients came to the emergency department after 3 h with a prehospital delay of 90.9% [26]. This research found that the majority came from urban areas. The analysis found that participants who were outside the Kampala district, the research site, had a higher prevalence of experiencing prehospital delays (98.7%) [26]. Research conducted in Bangladesh also found something similar with delays shown by most patients who used public transportation to reach the hospital [27]. For patients who live in rural areas, it was found that patients generally had first contact with a general practitioner or certified village doctor, which caused delays [27].

Research in Nepal found that all participants in the study said financial concerns were the biggest barrier to high-quality treatment. Participants mostly encountered patients who were unable or unwilling to pay for treatment. Therefore, most patients come to the hospital when their illness is in a serious stage. Handling patients in rural areas is more challenging. Apart from a lack of knowledge about health problems, there are differences in culture. People in rural areas generally seek traditional medicine first [28]. Rural areas often face longer delays due to limited access to healthcare facilities and emergency services. Additionally, rural populations may rely more heavily on traditional treatments before seeking medical care, further delaying hospital arrival. In contrast, urban areas generally have better access to healthcare resources, which can facilitate quicker response times and improved outcomes [28].

Baatiema et al. also stated that in conditions of low levels of public awareness of the early symptoms of stroke, healthcare practices are related to sociocultural conditions or religious beliefs [29]. Superstition is also an obstacle in patient management. Research in Ghana found that 25% believed that stroke was a spiritual disease caused by evil spirits or witches [30]. Stroke symptoms and signs are generally considered to be a spiritual consequence and must be dealt with spiritually. This has resulted in many patients being discharged from hospitals seeking herbal medicine or prayer camps and becoming more focused on spiritual life. Even in patients who are admitted to the hospital with a suspected diagnosis of stroke, misconceptions cause the patient not to take treatment, not to adhere to treatment, and to have problematic relations with healthcare professionals [29].

The level of education was found to be a critical determinant related to delays in seeking healthcare. Patients with a high school education or higher were less likely to experience delays. In immigrant populations, delays that occur may be caused by various things such as language barriers, cultural differences, lack of health insurance, and not understanding the local health system [31]. Superiority in educational level correlates with better general knowledge. The research found that even though the subjects in the research were mostly undergraduate level, this does not mean that appropriate emergency actions were taken immediately. The public still lacks knowledge regarding emergency actions that must be taken in cases of stroke [32,33]. This proactive behavior in seeking health help is not only the result of increased knowledge but also the result of extensive social networks and communities that facilitate access to assistance and guidance when there are health problems [34,35,36]. Variations in healthcare-seeking behaviors are built through the role of geographic, cultural, and socioeconomic factors [31,37].

**Table 2:** The sociodemographic and cultural barrier in low- and middle-income countries.

Challenges of Prehospital Delay in Stroke Care	Country
Knowledge of stroke warning signs and responses related to age, education level, hypertension status, and family history of stroke	Tanzania [25]
The majority of subjects only had primary education, and patients from outside the district experienced high prehospital delays	Uganda [26]
Patients in rural areas prefer to contact a general practitioner or village doctor first and use public transportation	Bangladesh [27]
Financial difficulty and cultural differences in rural areas lead patients to prefer traditional treatment	Nepal [28]
Cultural factors influence acceptance and compliance with treatment	Cameroon [38]
Illiteracy, primary education, living in a rural area, and no history of stroke are associated with low knowledge of stroke	Morocco [33]
Beliefs that strokes are caused by evil spirits or are spiritual problems lead patients to prefer traditional herbal medicine or faith healers	Ghana [30]

### 2.3. Healthcare System and Infrastructural Challenges

The health system and infrastructure also play a role in the treatment time of acute stroke, as summarized in Table 3. Stroke is in second place as a cause of death and is the top five cause of disability in the Philippines, a country with limited resources [39,40]. In the Philippines, there has been no translation of concrete steps regarding the stroke protocol development policy. Apart from this, access to diagnostic tools and drugs for stroke is limited, with EMS still fragmented [40,41]. In LMIC areas, the health system is generally fragmented and rarely has an organized stroke service program compared to high-income countries. In Ghana, there is no clear referral protocol to an adequate referral facility for treating stroke. Patients with stroke who come to the clinic are generally immediately admitted and only referred to a proper facility when the conditions deteriorate [42]. Research conducted by Gadama et al. found that in Malawi there is a large gap between international stroke guidelines and daily practice [43]. It was found that there was no consistent and structured implementation of guidelines for the management of patients with stroke. In addition, the absence of CT scans, important laboratory tests, and medications is the main reason for poor uptake of evidence-based practice and low implementation in Malawi [43]. This is also complicated by the national referral system which is still poor [43].

Ahadi et al. found that there was a gap in knowledge of stroke management among health professionals [19]. The study found that attitude scores towards stroke, knowledge of evidence-based management, and acute stroke management were significantly higher in nurses who participated in stroke retraining compared to EMS personnel [19]. Nurses who do not know about evidence-based services for stroke had a lack of participation in scientific programs related to stroke services [44,45,46]. Research finds that work experience, professional qualifications, and professional experience of stroke care in the workplace increase the efficiency of nurses in treating patients with stroke [47,48,49]. Mohammed et al. also said that handling acute stroke is less suitable for hospital emergency nurses who lack participation in training lessons [19,50]. Research also found that one of the causes of delay is related to referrals where patients contact community or local doctors first rather than going straight to the emergency room [20].

Transportation infrastructure such as ambulances is also important in some LMICs such as Somalia, where there is no emergency ambulance service [51]. Research conducted in Sierra Leone found that the median time from onset to patient arrival at the referral hospital center was 25 h with around 50.4% of patients seeking help first from other health service providers [52]. The time required for a patient from onset to arrival at the hospital if the patient goes directly to the referral hospital is 12.5 h, which is very different if patients seek services at other hospitals first with a time delay of around 51 h [52]. Potential alternatives such as the use of a prehospital mobile stroke unit and transcranial ultrasound may also improve stroke management in the prehospital setting [53,54,55,56].

**Table 3:** Healthcare and infrastructure barriers in low- and middle-income countries.

Challenges of Prehospital Delay in Stroke Care	Country
No concrete translation of stroke protocols due to limited resources, geographic barriers, and limited access to drugs and CT scans	Philippines [39,40,41]
No consistent and structured implementation of stroke guidelines for patients due to limited resources	Malawi [43]
Fragmented stroke services and unclear referral protocols lead to frequent delay	Ghana [42]
Personal EMS stroke management and attitude scores are higher in nurses with stroke retraining; EMS personnel need education in treating acute stroke	Bangladesh [27]
Work experience, stroke qualifications, and professional experience are related to nurse efficiency in treating stroke patients; EMS personnel need education and training in acute stroke services to improve patient outcomes	Iran, India [19,44,46,48,49]
Lack of emergency ambulance services	Somalia [51]
Delays due to patients seeking help from other health services before going to the referral hospital	Sierra Leone [52]

## 3. Diagnostic and Interventional Strategies

**Figure 2:**
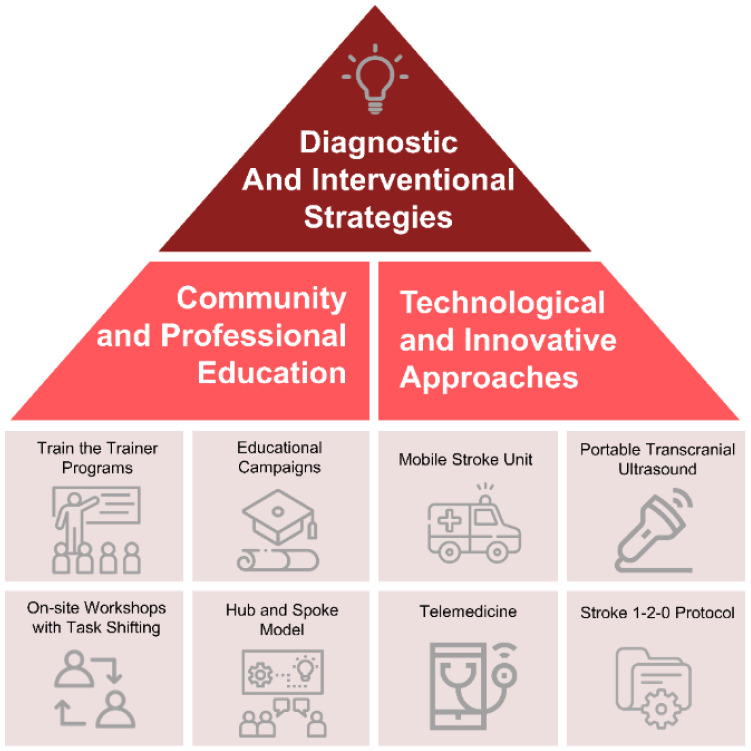
Diagnostic and interventional strategies for reducing prehospital stroke care delays in low- and middle-income countries. At the apex, diagnostic and interventional strategies encompass both community and professional education and technological and innovative approaches. The base of the pyramid details specific methods that correspond to their main category above it. These strategies are some examples from countries that collectively aim to enhance early diagnosis and timely intervention, bridging the gap in stroke care and improving patient outcomes in resource-limited settings.

To overcome the delays in providing stroke care in prehospital settings, it is necessary to develop creative diagnostic and interventional approaches that are specifically designed to address the limited resources available in LMICs. Prehospital systems in resource-limited areas such as rural communities face challenges in providing high-quality care. Currently, there is a new paradigm of bringing service to the patient rather than the patient to the service. Capability and number of human resources also influence acute stroke services. It is necessary to consider the local EMS system regarding staffing and availability of response to the community [57]. Technological progress and inventive strategies, such as mobile stroke units and telemedicine, provide encouraging alternatives to address the gap in stroke care. These tools enable prompt identification and intervention, potentially enhancing patient outcomes to a large degree [57,58]. Furthermore, both community and professional education play crucial roles in these methods. Targeted educational campaigns and training programs can enhance the knowledge and abilities of healthcare providers and the general public, leading to reduced delays and improved response times [59]. Some strategies discussed in this section have been drawn from developed countries to gain insights into the latest and existing solutions, as shown in Figure 2.

### 3.1. Use of Technological and Innovative Approaches

The use of technology is an example of a strategy that has been implemented in several LMICs to overcome the prehospital delay, as summarized in Table 4. One strategy to improve prehospital delay is the use of technology. Lilford et al. stated that ambulance services can provide open access for all “time-critical” emergency conditions [60,61]. One example of implementing a solution for this is the mobile stroke unit (MSU). Siriraj Hospital in Thailand became the first developing country to adopt an MSU for patients living in Bangkok and Nonthaburi. The MSU protocol is operated with two vehicles, a regular ambulance and an MSU [62]. Ordinary ambulances take patients to designated areas. Meanwhile, the MSU consists of doctors who have been trained in the protocol, stroke nurses, CT technicians, and drivers based at Siriraj Hospital. The MSU also has a CT machine and rtPA [62]. The study found that patients who were visited by an MSU were more likely to receive reperfusion therapy than the EMS group or the group who came alone. This study also found that patients who lived in underserved settings and received the MSU experienced faster door-to-needle processing times and had a higher probability of getting reperfusion. However, there was no significant difference between the groups regarding onset to door, which shows that there is a need to increase awareness. MSU can reduce delays for patients in remote areas who, if not using an MSU, would experience longer delays in arriving at the stroke center. Regarding costs, further studies are needed to assess the cost-effectiveness of an MSU compared to ordinary services, which have not been carried out in Thailand [62].

Regarding costs and infrastructure considerations, in 2008, a pilot study demonstrated the feasibility of using prehospital intracranial ultrasound to assess intracranial arteries in the prehospital setting [56]. Non-contrast enhanced transcranial arterial duplex ultrasound is performed by a neurologist on site or during the transfer of a potential stroke patient. Doppler visualization and flow in the middle cerebral artery can be assessed in 80% of patients, with the remainder being difficult due to the quality of the temporal bone window [56]. Recent studies have shown that transcranial ultrasound with a microbubble contrast agent can be used in cases of poor temporal bone windows [56]. Existing research on the use of portable ultrasound for early diagnosis in emergency settings is starting to increase but is still not considered a standard tool for prehospital personnel [56]. The use of wireless technology and telemedicine can be an alternative. Real-time assessment by a neurologist using telecommunications technology can improve stroke services [63,64]. Time is an important part of prehospital care. Emergency medical responders need to work quickly and efficiently to provide critical services to patients. With time being a critical factor, emergency medical responders need to be able to work under pressure and prioritize their actions to maximize positive outcomes. To overcome this concern, strict protocols are required to ensure that prehospital care providers have appropriate training and education [65]. Liu et al. stated that general practitioners play the role of gatekeepers in community health [66]. Previous research explains that stroke-related education among general practitioners can increase community knowledge regarding stroke risk factors [66].

In 2016, Zhao et al. proposed the 1-2-0 stroke identification rule for early identification: “1” to check if the corners of the mouth are skewed, “2” to check if there is unilateral limb paralysis, and “0” to listen to see if there is slurred speech [67]. If symptoms arise, the general practitioner needs to immediately call 120 as the emergency number [67]. Liu et al. conducted training for general practitioners in five communities regarding the 1-2-0 protocol [66]. After training, doctors better understood the time window for intravenous thrombolysis, and most community doctors immediately called the emergency number, thereby shortening prehospital delays. There was more use of the emergency system and an increased rate of thrombolytic therapy after training, and the arrival time of patients with stroke within 3 h increased up to three times [66]. In the past, stroke education in community residents was generally provided by specialists, and most educated individuals soon forgot due to a lack of repetition of information, causing a lack of knowledge retention. Education via social media is generally expensive and requires continuous consumption of quite large resources and does not directly provide educational effects. Meanwhile, stroke education focused on the community environment is cheaper and also improves the relationship between neurologists and general practitioners [66].

**Table 4:** The example of technological and innovative approaches for overcoming stroke prehospital delay.

Innovative Approach	Country	Summary	Advantage	Disadvantage
Mobile stroke unit	Thailand, Germany [58,62,68,69]	Ambulance with portable CT scan, lab, and stroke treatment to reach patients quickly	Speeds up diagnosis and treatment	Expensive, complex, needs multi-professional resources
Portable transcranial ultrasound	Germany [56]	Uses ultrasound to visualize arteries and aid diagnosis	Cheaper costs	Requires trained experts, more research needed
Telemedicine	Germany [63,64]	Wireless technology for real-time video streaming from ambulance to assist in diagnosis	Increases access to experts, reduces geographic barriers	Needs good infrastructure and signal
Stroke 1-2-0	China [66,67]	Early stroke identification for use by general practitioners, increasing awareness and reducing delays	Early detection and rapid response, educational tool	Needs further research in diverse locations and populations

### 3.2. Community and Professional Education

As shown in Table 5, various educational approaches have been implemented to address prehospital stroke delays in LMICs. Menon et al. found that stroke awareness has not made substantial progress in the last two decades [70]. Multivariate analysis found that education level was the only variable that correlated with stroke recognition, organ identification, and danger sign identification [70]. Mishra et al. also found delays related to patient knowledge, so there was a need to increase community awareness [71]. Although knowledge of stroke symptoms and risk factors as well as the location of stroke units is high, the public still does not understand the steps that should be taken when a stroke occurs [32]. Research in China found that only 23.3% were aware of therapy for acute ischemic stroke. There is a need for public education that educates the public about thrombolysis therapy. Existing studies show that half of respondents get stroke information from television and newspapers [72].

Stroke education campaigns are needed because of the importance of early recognition and rapid response to stroke symptoms and realizing the importance of access to thrombolysis as therapy. Education with a combination of 3 months of mass media campaigns in the community and for health professionals was related to an increase in the utilization of thrombolysis. The number of stroke patients coming to the hospital within 24 h increased from 37% to 86% [73,74]. Another study also implemented a 15-month intervention targeting communities and professions to increase the proportion of patients receiving thrombolysis. This study found that in the intervention group, there was an increase in thrombolysis treatment, and the improvement in management persisted until after the intervention, thus explaining that the effect was primarily on the health profession rather than the community [74,75].

Research shows that public education requires appropriate targets such as stroke survivors as well as the patient’s family and neighbors [74]. Stroke education can also be given to health students and children as a medium for conveying information. Messages need to be simple and carried out in appropriate locations such as places of worship and waiting rooms using appropriate media. Continuous exposure is necessary to achieve success [74]. Education also needs to target ethnic minorities and the elderly because symptoms can cause patients to be unable to perform a phone call. Apart from that, campaigns require effective instruments such as video, music, multimedia, and social media, as well as appropriate platforms such as schools and places of worship, and they need to explain what needs to be done and be repetitive like commercials [76].

A systematic review carried out by Habibi et al. determined that various educational interventions have the potential for positive results in stroke care education in low-resource locations [59]. “Train the trainer” is the most potential technique with promising technology [59]. “Train the trainer” uses a doctor who is trained by a local expert for 2 h via video conference [59]. The training is then built into the implemented health protocols. After implementation, it was found that there was an increase in re-education and mobility assessment, outcomes were also better, and complications decreased [59,77]. Education seems to have a positive effect on the desired outcomes in developing a stroke unit [59]. Another educational technique is an on-site workshop with task-shifting education for strategies for using existing human resources efficiently. Tasks initially carried out by doctors are transferred to other health professions with training for limited work only. The one-day workshop provides information on stroke from risk factors to management to health officials, nurses, and community health workers. Presentations are given in didactic, documentary, and interactive or practical sessions. After doing this, there was an increase in the use of thrombolytics including knowledge of risk factors and symptoms [59,78]. Multidimensional education carried out in Brazil, Argentina, and Peru uses case management, roadmaps, checklists, educational materials, audits, and feedback [79]. The use of technology is carried out in Brazil by using technology-based hubs (tertiary health care centers) and spokes (five emergency departments) to improve stroke diagnosis accuracy and quality. Neurologists from the hub provide training to spoke facility department staff connected by telemedicine technology [59,80].

**Table 5:** The example of community and professional education for overcoming stroke prehospital delay.

Educational Approach	Country	Summary	Advantage	Disadvantage
Educational Campaign Intervention with Mass Media	Germany, India Saudi Arabia, China [32,70,72,74,76]	Campaigns to educate about stroke symptoms, increasing alteplase treatment rates	Increases public knowledge and appropriate response to stroke	Varies in success rate, needs further research
Train The Trainer Intervention	India, Ghana, and China [59,77]	Training general practitioners by local experts via video conferencing to implement protocols	Improves doctors’ ability to manage stroke and trains leaders	Requires comprehensive multidisciplinary training and good protocols
On-site workshop with task-shifting	Nigeria [59,78]	Training general practitioners for stroke treatment by specialists	Efficiently increases human resources in underserved areas	Needs research on knowledge retention and success rates
Technology for education	Brazil, India, China [59,80,81,82]	Telemedicine to connect experts with underserved areas; mobile apps for diagnosis and treatment	Connects experts with areas lacking specialists, aids in management and education	Requires adequate telemedicine infrastructure

## 4. Case Studies and Pilot Programs

**Figure 3:**
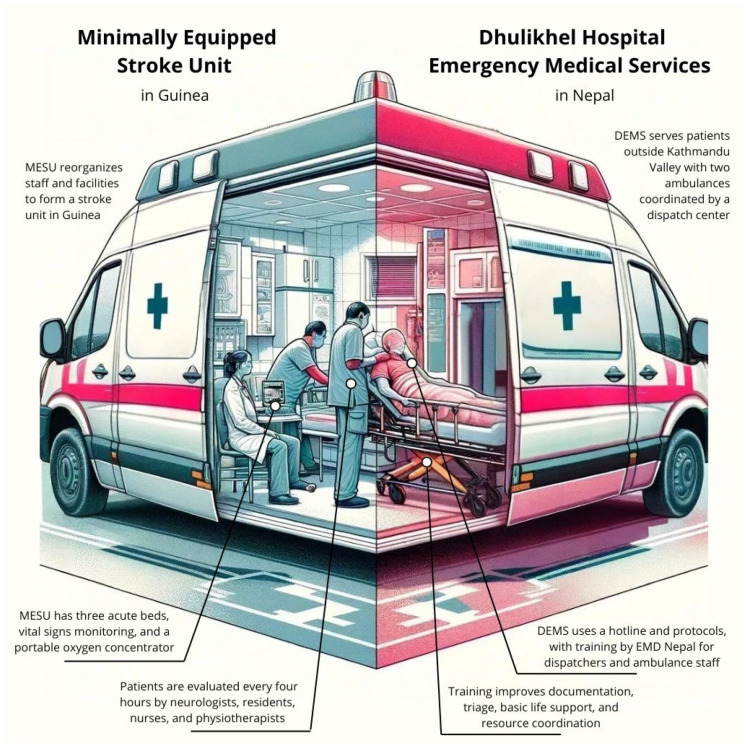
Case studies of emergency medical services in low- and middle-income countries. On the left, the minimally equipped stroke unit (MESU) in Guinea reorganizes existing staff and facilities to form a functional stroke unit, featuring three acute beds, vital signs monitoring, and portable oxygen concentrators. Patients are evaluated every four hours by neurologists, residents, nurses, and physiotherapists. On the right, Dhulikhel Hospital Emergency Medical Services (DEMS) in Nepal serves patients outside Kathmandu Valley with two ambulances coordinated by a dispatch center. DEMS uses a hotline and protocols, as well as training provided by EMD Nepal for dispatchers and ambulance staff, which enhances documentation, triage, basic life support, and resource coordination. These case studies demonstrate practical strategies to improve stroke care and reduce prehospital delays in resource-limited settings.

Studying efficient methods and pilot initiatives offers valuable insights into practical strategies for overcoming stroke care obstacles in LMICs. Case studies illustrate the successful implementation of innovative tactics and their positive outcomes, as shown in Figure 3. These examples provide evidence-based methods that can be adapted and scaled in similar settings. Additionally, this analysis highlights the impact of training programs on improving the proficiency of healthcare professionals and EMS personnel, emphasizing the necessity of continuous education and skill enhancement.

### 4.1. Dhulikhel Hospital Emergency Medical Services in Nepal

The challenge of providing effective prehospital care in LMICs is multifaceted, encompassing issues like insufficient staffing, inadequate ambulance availability, and the high costs associated with ambulance services. One of the problems of LMICs is the lack of staff, lack of ambulances, and high costs of ambulance services. Jacobson et al. try to address this challenge in Dhulikhel Hospital, Kathmandu [83]. They implemented Dhulikhel Hospital Emergency Medical Services (DEMS), which aims to bring prehospital services to patients outside the Kathmandu Valley [83]. Dhulikhel Hospital has only two ambulances in the system, and the rest are coordinated by the dispatch center but owned by the local government [83]. This system has a hotline number of 102. Dispatch protocol employs high-income countries to design a good integrated network for communication through the system. Using an international dispatch system is thought to be financially unsustainable and not applicable to infrastructure and decentralized hospitals in Nepal. Therefore, training and evaluation materials were created directly by EMD Nepal. Training is also provided for emergency medical dispatchers or ambulance staff, both paramedics and first responders who may receive calls from the community. Apart from knowledge training, scenario-based skill assessments are also carried out [83]. Pilot training is provided to all EMS dispatchers who have never received training about ambulance dispatch before. In this research, it was found that all dispatchers got a score above the passing score for scenario-based skill evaluation, with only one participant needing to retest. After training, all dispatchers stated that the implementation of dispatcher training had a positive impact on the DEMS system. After training with protocols, dispatchers began to document all activities carried out. Reliable documents can help improve dispatcher quality and accountability. Dispatchers can also conduct triage and basic life support, as well as pre-notify hospitals, thereby closing communication gaps. Training also increases ambulance resources across locations based on better triage and coordination. This training is a resource-efficient, innovative, and cost-effective strategy to strengthen prehospital care in LMICs [83].

### 4.2. Minimally Equipped Stroke Unit in Guinea

Imaging facilities and stroke management centers are an exception in LMIC due to accessibility and cost parameters. This is where the stroke unit comes into play. The stroke unit is organized inpatient care provided by a multidisciplinary team consisting of medical, nursing, physiotherapy, speech therapy, and social worker staff. This stroke unit aims to improve diagnosis and management, prevention of complications, and appropriate monitoring. Evidence shows that stroke patients have better outcomes when treated in stroke units. Therefore, stroke units can be an option for LMICs. In an ideal stroke unit, it is necessary to have nurses trained for stroke, CT scans available within 24 h that are prioritized for stroke patients, extracranial Doppler ultrasonography, automatic electrocardiography monitoring, and rt-PA protocols. However, not everything can be fulfilled in LMIC. Therefore, a minimally equipped stroke unit (MESU) can be applied [84].

In a study in Guinea, it was found that mortality was significantly reduced in patients treated at an MESU [85]. In Guinea, the implementation of minimum stroke unit settings was carried out in research at Ignace Deen Hospital. Guinea itself is a sub-Saharan country that is among the poorest countries in the world. CT scans are only available in one national hospital out of the three existing national hospitals. In addition, two-thirds of healthcare costs are paid by patients, making them inaccessible to more than 40% of the population. The MESU has three acute beds separate from the neurology ward and has vital signs monitoring and a portable oxygen concentrator. Patients are evaluated every 4 h regarding clinical parameters, body temperature, and NIHSS carried out by neurologists, residents, nurses, and physiotherapists [86]. Reorganization of existing staff and facilities for multidisciplinary services can improve outcomes without increasing resource use [85].

## 5. Future Directions and Innovations

**Figure 4:**
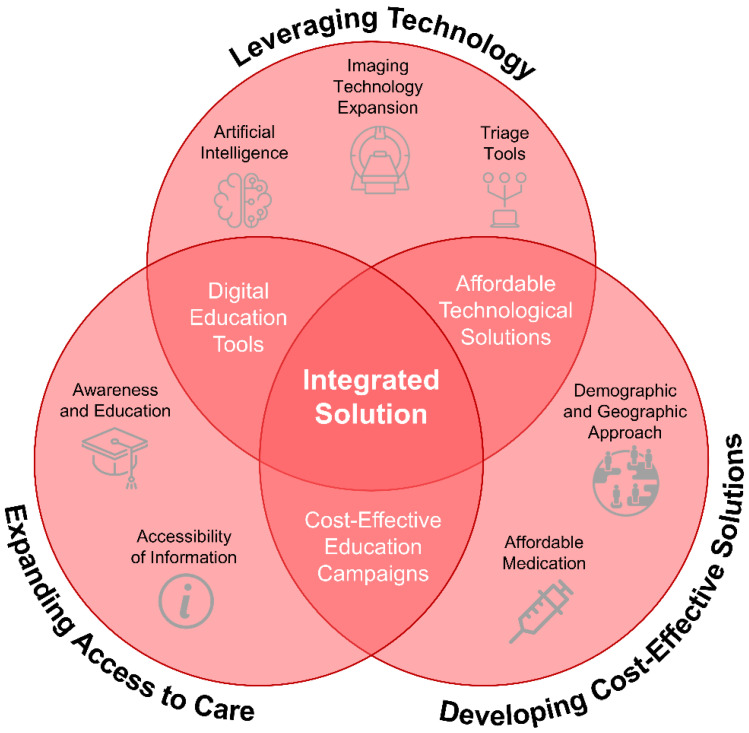
Future directions and innovations in prehospital stroke care for low- and middle-income countries. This Venn diagram highlights three primary strategies: leveraging technology (including imaging technology expansion, triage tools, and artificial intelligence), expanding access to care (focusing on awareness and education, as well as accessibility of information), and developing cost-effective solutions (such as affordable medication and demographic and geographic approaches). At the intersection of these strategies are digital education tools, affordable technological solutions, and cost-effective education campaigns, forming an integrated solution aimed at enhancing stroke care outcomes in resource-limited settings.

In developed countries with adequate resources and finances, prehospital stroke services already have standard protocols with the aim of accelerating treatment. Medical and paramedic teams are well trained, and stroke education is always regularly provided to the community. Stroke services are also generally clearly regionalized with clear referral hospitals for stroke cases. Innovation and research are also ongoing and implemented, such as mobile stroke units and telemedicine [76,87]. Meanwhile, the future of prehospital stroke care in LMICs involves developing cost-effective strategies, increasing care availability, and leveraging technology. By examining previous challenges and effective approaches, we can address the unique needs of these regions. This includes tackling financial constraints and limited healthcare facilities, as shown in Figure 4. Advances in medical technology, such as portable diagnostic devices and artificial intelligence, offer significant opportunities for early detection and intervention. This section summarizes potential future trends and examines probable breakthroughs and their implications for stroke care in LMICs.

### 5.1. Developing Cost-Effective Solutions

Several cost-effective solutions can be alternatives in reducing prehospital delays. Research in India found that gender and location of residence influenced prehospital delay [88,89]. Advanced age and female gender are correlated with prehospital delay. Gender may have an impact due to the absence of family members to help with transportation and many women being home alone or having children. Regarding location of residence, living in an urban area is a significant factor in arriving early at the hospital. This could be due to more information obtained by patients and family members and more effort from emergency service teams throughout the city. Therefore, there is a need for solutions that target specific attention to rural areas and sociodemographic factors [88,89]. If we refer to high-income countries, it is difficult to adopt standard neurocritical management in almost all LMICs. The gap occurs due to multifactorial factors, such as before implementing advanced modalities, fundamental resources are needed first, such as readily accessible neuroimaging, nurses with neurology expertise, medicines, and others. In resource-limited settings, duplication of practice in HICs will be challenging and may not result in improved outcomes. Therefore, efforts are needed to build capacity by identifying challenges, and longitudinal efforts are needed from health workers to improve health service systems in LMICs [90].

Another barrier to LMIC prehospital delay is the economic and high cost of rt-PA, which is an important treatment for acute ischemic stroke. However, there is another type of drug, namely, Tenecteplase, which can be an alternative. Tenecteplase was found to have a longer duration of action and can be given as a single bolus. The study also showed that 78.5% of those given Tenecteplase experienced a decrease in their NIHSS score within 24 h and only one subject experienced symptomatic intracranial bleeding and died. Apart from being safe and having efficacy in stroke, the cost of Tenecteplase is almost half the cost of alteplase, so it can be an alternative in resource-limited settings [91,92,93,94].

### 5.2. Expanding Access to Care

Research conducted in Botswana found that there was a low level of awareness of seeking or calling EMS in acute stroke conditions [95]. This study found that outpatients had a higher level of awareness than the public to call EMS in the acute stroke setting [95]. Research in Ghana found that the majority of patients in the study had primary-level education [96]. Only 16% understood that the symptoms they were experiencing were a stroke before arriving at the hospital, and there were a large number of patients who refused or could not decide regarding thrombolysis due to cost inability or due to doubts about its effectiveness [96]. This shows the need for extensive educational strategies in LMICs [96]. Research in Indonesia found that 77.5% of patients had a low level of awareness of stroke [97]. Low levels of awareness influence delays in visiting health centers. This research shows that in the community, the level of awareness of stroke is still insufficient. This shows the need for disseminated stroke education and stroke awareness campaigns, especially for patients with stroke risk factors [97].

Existing research also shows the need to increase the accessibility of health and education service information. Education is needed, especially regarding actions that must be taken when experiencing an acute stroke. Bearing in mind that even though knowledge about stroke is good, the public still does not understand the steps that must be taken when experiencing an acute stroke [17,32]. Research also finds that mass media such as TV, newspapers, and family and friends are the highest distributors of stroke information. Awareness to call EMS or seek medical help is helped by almost 70% due to information conveyed from various methods and sources [95].

### 5.3. Leveraging Technology

Aguirre et al. emphasized that imaging technology such as CT and MRI are needed in the management of patients with stroke [1]. Doctors use CT or MRI to diagnose the condition and to determine therapy. This indicates the need for the expansion of imaging availability, the possible need for portable diagnostic tools, and the application of telemedicine technology to assist in early stroke diagnosis in the prehospital setting [1]. In optimizing stroke services, triage plays an important role. After symptoms and a call for help, EMS will come to the patient’s location. The EMS team will triage and determine patients with stroke and take them to the nearest stroke center. There are several tools that EMS teams can use, ranging from simple clinical stroke severity scales to mobile stroke units [98].

Clinical stroke severity scales such as Face-Arm-Speech-Time (FAST) or Rapid Arterial oCclusion Evaluation (RACE) are the simplest pre-hospital triage tools and do not require specialized equipment and do not require additional costs to the system beyond basic training for personnel [98,99]. The use of this triage also improves pre-hospital management and shortens preparation for endovascular thrombectomy. Although some pre-hospital stroke severity scales are dichotomous, most are more complex than dichotomous scores and have varying cut-offs and variations in sensitivity and specificity [98,99]. RACE, G-FAST (Gaze-Face-Arm-Speech-Time), and CG-FAST (Conveniently Grasped Field Assessment Stroke Triage) have the best performance with similar accuracy to the NIHSS (National Institutes of Health Stroke Scale) performance of various prehospital triage scales that vary across the spectrum of deficit severity [100]. In patients with intermediate severity symptoms with uncertainty in the diagnosis, the performance of the clinical symptoms severity scale is very low. The most important thing in performance using a stroke scale is that EMS personnel need to be familiar with and comfortable using it. Apart from this, several applications can help MESUs in determining pre-hospital severity assessment and risk stratification [98,101,102]. Another innovation that could be an alternative is the use of artificial intelligence (AI) to determine decisions in the prehospital setting. AI is a broad term in computer science and denotes computers that perform tasks that are considered difficult for humans. Machine learning is a part of computer AI for solving problems based on data applied to giant datasets. AI has the potential to determine patient management and therapy as well as assist in accurate and rapid stroke characterization. This can be helped by the development of a computerized decision support system (CDSS) based on machine learning algorithms. AI-based CDSS is still the focus of development to help treat prehospital and rural areas [103].

## 6. Limitation

One of the primary limitations of this review is the scarcity of studies specifically addressing the risk factors for prehospital delay in low- and middle-income countries (LMICs). Due to this limitation, it was challenging to conduct a specific review for some countries. We have ensured that we conducted a comprehensive and robust search for relevant papers and have summarized the findings in tables within each sub-section. However, some countries do not publish data on these factors. We believe these factors do exist in practice, but since we are conducting a literature review, we have only included data based on published literature, not opinions. This limitation is recognized and addressed in the limitations paragraph of our paper.

Another limitation is the availability of data on door-to-needle times, which was not consistently reported across all countries. While we have included these data where available, the lack of uniform reporting standards makes it difficult to provide a comprehensive comparison. We recognize that these factors likely exist in practice, but since this is a literature review, we have only included data based on published studies. This limitation underscores the need for more research and better reporting standards in LMICs.

Additionally, our review primarily discusses stroke in general, as most studies do not differentiate in detail between the prehospital management of ischemic and hemorrhagic strokes. In-hospital management clearly distinguishes between the two, with different treatments required for each type. Throughout our manuscript, we have identified sections specific to ischemic or hemorrhagic strokes when applicable. However, it is important to note that most studies focus more on ischemic strokes, which is a limitation of our review.

## 7. Conclusions

The existing literature identifies several causes of prehospital delays in stroke care, including patient awareness and knowledge, socioeconomic and cultural factors, and health system and infrastructure challenges. Addressing these delays requires a multifaceted approach that integrates comprehensive education, technological advancements, and innovative solutions tailored to the specific needs of low- and middle-income countries (LMICs). Effective interventions in LMIC settings must be cost-efficient, enhance access to education and healthcare services, and leverage technology to streamline processes. The ultimate goal is to reduce prehospital delays, thereby improving the efficiency and effectiveness of acute stroke care services.

While this study emphasizes stroke incidence and prehospital care in LMICs, comparing these approaches with those in developed countries provides valuable insights. In developed countries, higher levels of public awareness and education about stroke symptoms lead to quicker response times. Comprehensive emergency medical services (EMS) are more widely available and better coordinated, resulting in shorter door-to-needle times. Additionally, healthcare infrastructure in developed countries is more robust, with widespread availability of advanced diagnostic and treatment facilities. In contrast, LMICs face significant challenges in these aspects. Public awareness campaigns are less prevalent, and cultural beliefs can delay seeking medical care. EMS systems may be fragmented or under-resourced, leading to longer response times and delays in treatment. Healthcare infrastructure in LMICs often lacks the necessary resources and facilities to provide timely and effective stroke care.

By examining these differences, LMICs can identify areas for improvement. Implementing public education campaigns to increase awareness of stroke symptoms, investing in EMS systems, and enhancing healthcare infrastructure are critical steps that can be informed by successful strategies employed in developed countries. Adopting cost-effective technologies and innovations can also help bridge the gap and improve prehospital stroke care in LMICs. By fostering community awareness, enhancing healthcare infrastructure, and implementing scalable technological innovations, LMICs can make significant strides in reducing the burden of stroke-related morbidity and mortality.

## 8. Methods

This study is a comprehensive literature review aimed at summarizing the current knowledge, challenges, and potential solutions for prehospital stroke care in low- and middle-income countries (LMICs). A systematic search was conducted in multiple databases, including PubMed, Scopus, Web of Science, Cochrane Library, and Google Scholar. The search terms included “Prehospital stroke care”, “Stroke management in LMICs”, “Stroke awareness”, “Emergency medical services”, “Thrombolytic therapy”, “Mobile stroke units”, “Telemedicine in stroke care”, and “Public health education”. There were no language restrictions, and studies from both low- and high-income countries were included to provide a comprehensive perspective.

We included articles published in peer-reviewed journals that focused on prehospital stroke care, discussed challenges and solutions related to stroke care, and provided relevant data or insights. We excluded articles that were not related to stroke care; were focused solely on high-income countries without providing transferable insights to LMICs; or were non-peer-reviewed articles, editorials, or opinion pieces.

The initial search yielded 1917 articles. After screening for relevance and applying the inclusion and exclusion criteria, 149 articles were selected for detailed review. The final selection comprised 89 articles that provided comprehensive insights into the challenges and solutions in prehospital stroke care in LMICs. The review identified several key challenges, including lack of awareness, sociodemographic and cultural factors, and healthcare system limitations. Innovative solutions such as mobile stroke units, telemedicine, and targeted educational campaigns were discussed.

## Data Availability

All data that were analyzed have been presented in this study.
